# Antibiotic prescribing patterns in patients hospitalized with COVID-19: lessons from the first wave

**DOI:** 10.1093/jacamr/dlab085

**Published:** 2021-06-30

**Authors:** Brendan O’Kelly, Colm Cronin, David Connellan, Sean Griffin, Stephen Peter Connolly, Jonathan McGrath, Aoife G Cotter, Tara McGinty, Eavan G Muldoon, Gerard Sheehan, Walter Cullen, Peter Doran, Tina McHugh, Louise Vidal, Gordana Avramovic, John S Lambert

**Affiliations:** 1 Infectious Diseases Department, Mater Misericordiae University Hospital, Dublin 7, Ireland; 2 Centre for Experimental Pathogen Host Research, University College Dublin, Dublin 4, Ireland; 3 School of Medicine, University College Dublin, Dublin 4, Ireland

## Abstract

**Background:**

A high proportion of hospitalized patients with COVID-19 receive antibiotics despite evidence to show low levels of true bacterial coinfection.

**Methods:**

A retrospective cohort study examining antibiotic prescribing patterns of 300 patients sequentially diagnosed with COVID-19. Patients were grouped into 3 sub-cohorts: Group 1 received no antibiotics, Group 2 received antibiotics for microbiologically confirmed infections and Group 3 was empirically treated with antibiotics for pneumonia. The primary aim was to identify factors that influenced prescription and continuation of antibiotics in Group 3. Secondary aims were to examine differences in outcomes between groups.

**Results:**

In total, 292 patients were included (63 Group 1, 35 Group 2, 194 Group 3), median age was 60 years (IQR 44–76) and the majority were ethnically Irish (62%). The median duration of antibiotics was 7 days (IQR 5–10). In Group 3, factors associated with prescription IV antibiotics on admission were raised C-reactive protein (CRP) (*P *=* *0.024), increased age (*P *=* *0.023), higher quick SOFA (*P *=* *0.016) score and fever >37.5 °C (*P *=* *0.011). Factors associated with duration of antibiotic course were duration of hypoxia (*P *<* *0.001) and maximum respiratory support requirement (*P *=* *0.013). Twenty-one patients in Group 3 had one or more antibiotic escalation events, most (*n *=* *139) had no escalation or de-escalation of therapy.

**Conclusions:**

Duration of hypoxia and need for respiratory support may have acted as surrogate measures of improvement where usual response measures (CRP, neutrophilia, culture clearance) were absent. Continuous review of antibiotic prescriptions should be at the forefront of clinical management of hospitalized patients with COVID-19.

## Introduction

An initial lack of informed guidance on antimicrobials since the beginning of the COVID-19 pandemic has led to broad range of antimicrobials being used in those hospitalized with COVID-19.^1^ Former experience informed by influenza virus epidemics shows early infection with *Staphylococcus aureus, Streptococcus pneumoniae* and *Haemophilus influenzae* can be severe and have increased mortality in influenza patients.^2^^,^^3^ Given a poor understanding of the rate of bacterial coinfection during the first wave of COVID-19, use of antibiotics in these acutely unwell patients was not entirely unreasonable at that time. It has subsequently borne out that the rate of true bacterial infection with COVID-19 has been at levels of around 7%–8%.^4^^,^^5^ Nevertheless, the impact of COVID-19 on antimicrobial resistance may be manyfold, not only the effect of increased prescribing but the direct impact of COVID-19 on the ability of antimicrobial stewardship (AMS) services to be impactful in their role. One online survey of 86 respondents working in AMS found a significant decrease in the mean impact of their AMS services during the COVID-19 era.^6^ The impact of increased antimicrobial prescribing during the COVID-19 pandemic and the impact on antimicrobial resistance (AMR) have yet to be fully discerned.^7^^,^^8^

To date there are many studies that describe antibiotic consumption in hospitalized patients with COVID-19 and the mismatch between high levels of antimicrobial use and low levels of proven bacterial coinfection.^4^^,^^9^^,^^10^ Almost none describe the individual factors associated with commencement, continuation, escalation, de-escalation or discontinuation of antibiotics in COVID-19 patients. Identifying patterns and factors associated with empirical antimicrobial prescriptions in the context of COVID-19 is key to informing current and future AMS services.

The primary aim of this study is to examine factors associated with antimicrobial prescribing (commencement, escalation, de-escalation and overall duration) in patients hospitalized with COVID-19 treated empirically for pneumonia. These prescribing patterns may give us some insight into prescribing behaviour. Secondary aims include comparing outcomes of the three subgroups: those that did not receive antibiotics, those that received antibiotics for proven bacterial coinfection and those that received antibiotics empirically (the group described in the primary aim).

## Methods

### Study outline

This study was a retrospective, single-centre cohort study examining hospitalized patients with confirmed COVID-19 by nasopharyngeal PCR (NP -PCR) testing. The study was conducted in Mater Misericordiae University Hospital (MMUH) Dublin, a 580 bed hospital containing Ireland’s National Isolation Unit (NIU). Participants were included if they had confirmed COVID-19 on NP-PCR, were ≥18 years of age, and were hospitalized. Patients were excluded if insufficient data were collected for meaningful analysis. The first 300 consecutive confirmed COVID-19 infections were enrolled in the study. Dates of admission were between 9 March and 28 May 2020. Patients were grouped into three cohorts: Group 1—patients who did not receive antibiotics; Group 2—patients who received antibiotics for confirmed bacterial infection by microbiological sampling and/or non-respiratory infectious complications like osteomyelitis and cellulitis; and Group 3—patients who received empirical antibiotics for undifferentiated pneumonia in which uncomplicated bacterial pneumonia was suspected or was not ruled out.

In Group 2, true infection was considered if patients had clinical features plus laboratory, radiological or microbiological samples supportive of bacterial infection. Patients categorized into this group include central venous catheter (CVC) infections, hospital-associated pneumonia (HAP) or ventilator-associated pneumonia (VAP), urinary tract and catheter-associated infection, bacteraemia, osteomyelitis, and abscess with supportive microbiology. Patients that were treated empirically for infections other than pneumonia like cellulitis, urinary tract infection or other infections where an organism was not identified were also included in this group. The aim of generating this grouping is to filter out these patients from the larger Group 3 patients who received empirical antibiotics for pneumonia where prescribing patterns were not guided by microbiology or other confirmatory investigations.

At the onset of the first wave anecdotal evidence for the efficacy of azithromycin in combination with hydroxychloroquine from small and flawed studies led to the widespread use of this combination as a potential COVID-19 directed therapy. As azithromycin was prescribed for this purpose and not for its antibacterial properties it was excluded from group analyses as it offers little insight into prescribing patterns for bacterial pneumonia. Azithromycin was only prescribed with hydroxychloroquine for COVID-19 patients with presence of pulmonary infiltrates and/or for the need of supplemental oxygen. Cognizance of potential stock shortages resulted in it not being prescribed solely as atypical pneumonia coverage: clarithromycin or doxycycline were used for this purpose.

### Data control

The data was extracted from the Anticipate study database and work is permitted under the approval of this study.^11^ The Anticipate study is a longitudinal study examining outcomes in COVID-19 patients funded by the Health Research Board, Ireland. Data of pseudo-anonymized patients was collected on a password encrypted database in Microsoft Excel^®^ 2019 and stored on a shared drive on the firewall protected hospital server. All data collection and access to data was performed by members of the research team.

### Parameters recorded

Baseline demographics recorded were sex, age, suspected environment of acquisition, comorbidities, duration and nature of symptoms. Severity markers included vital signs [respiratory rate per minute (RR); heart rate per minute (HR); maximum temperature in Celsius scale (Tmax); systolic and diastolic blood pressure in mmHg (SBP/DBP); oxygen saturation (SpO_2_)] and Glasgow Coma Scale (GCS) was also recorded. A quick SOFA (qSOFA) score was retrospectively calculated using RR, GCS and SBP. qSOFA is a well-validated tool for predicting adverse outcomes in patients admitted with sepsis; a score of ≥2 has been shown to have 3 to 14 times increased in-hospital mortality.^12^ Laboratory parameters recorded were lymphocyte count, neutrophil count, ALT, ferritin, d-dimer and troponin level. Radiological findings from X-ray and CT were documented and categorized into three groups: (i) normal imaging; (ii) findings consistent with pneumonitis; and (iii) other changes including unilateral pneumonia and atelectasis. Treatment measurements were also collected from paper-chart records; high-level supplemental oxygen (high-flow oxygen, non-invasive ventilation, mechanical ventilation), antimicrobial route and duration, and COVID-19 directed therapy. Outcome measurements included intensive care admission, length of stay (LOS), complications, readmission and mortality.

### Statistical analysis

IBM SPSS Statistics v.24.0 (IBM Corp., Armonk, NY, USA) was used for descriptive and inferential statistical analysis. Continuous data are presented as median and IQR. Kolmogorov–Smirnov analysis was used to test for normality testing. The χ^2^ test was used for categorical dependent and independent variables. The Mann–Whitney *U*-test was performed on non-normally distributed nominal data with two groups, Kruskal–Wallis was used for non-normally distributed data of >2 groups. Dichotomous linear regression and multivariable linear regression were used when the dependent variables were dichotomous or continuous, respectively. A *P* value <0.05 (two-tailed) was deemed to be statistically significant.

### Ethical approval

The project received approval from the Master Misericordiae University Hospital Research Ethics Committee on 8 April 2020 (reference 1/3782141).

### Availability of data/material

Anonymized FAIRifed study data will be made available upon publication of this research manuscript. We anticipate that COVID-19 study data will be uploaded to ZENEDO data repository under a persistent identifier.

## Results

Of 300 patients initially enrolled, 8 were excluded due to a lack of data. The remaining 292 were included in the final analysis. A majority of patients were male *n *=* *154 (52.7%) and ethnically Irish *n *=* *181 (62%), with a median age of 60 years (range 17–97), The most common comorbidities were hypertension *n *=* *90 (30.8%), dyslipidaemia *n *=* *55 (18.8%) and cognitive impairment *n *=* *47 (16.1%). Comorbidities aligned with poor outcomes in COVID-19 like diabetes mellitus and obesity were seen in 45 (15.4%) and 14 (4.8%) of patients, respectively. Other comorbidities, symptoms, initial severity markers, radiology and laboratory findings on admission can be seen in Table 1.

**Table 1.** Patient demographics

Demographic	*N* (%) or median	IQR
Total	292	
male	154 (52.7)	
female	138 (47.3)	
Age	60	44–76
Nationality		
Irish	181 (62)	
non-Irish	68 (23.3)	
unknown	43 (14.7)	
Nursing home resident	51 (17.5)	
Suspected mode of acquisition		
community associated	199 (68.1)	
unknown source	133 (45.5)	
household contact	37 (12.7)	
travel related	16 (5.6)	
non-healthcare occupational	13 (4.5)	
healthcare associated	93 (34.9)	
nursing home resident	50 (17.1)	
occupational	27 (9.2)	
other^a^	16 (5.5)	
Duration of symptoms pre diagnosis		
<48 h	65 (22.3)	
2–7 days	140 (48)	
>7 days	70 (24)	
unknown	17 (5.8)	
Symptoms		
cough	201 (68)	
fever	179 (61.3)	
shortness of breath	133 (45.5)	
fatigue	94 (32.2)	
myalgia	67 (22.9)	
headache	47 (16.1)	
sore throat	38 (13)	
diarrhoea	34 (11.6)	
nausea/vomiting	34 (11.6)	
chest pain	34 (11.6)	
decreased level of consciousness	22 (7.5)	
abdominal pain	13 (4.5)	
loss of taste	9 (3.1)	
rhinorrhoea	8 (2.7)	
seizure	1 (0.3)	
Admission severity markers		
quick SOFA (qSOFA)		
0	156 (53.4)	
1	113 (38.7)	
≥2	22 (7.5)	
Comorbidities		
any chronic illness	200 (68.5)	
any cardiac disease	125 (42.8)	
hypertension	90 (30.8)	
any respiratory illness	70 (24)	
dyslipidaemia	55 (18.8)	
cognitive impairment	47 (16.1)	
diabetes mellitus	45 (15.4)	
ischaemic heart disease	41 (14)	
psychiatric diagnosis	38 (13)	
chronic kidney disease	35 (12)	
asthma	34 (11.6)	
COPD	29 (9.9)	
osteoarthritis	29 (9.9)	
malignancy (current or prior)	23 (7.9)	
current	9 (3.1)	
prior	7 (2.4)	
haematological	7 (2.4)	
congestive cardiac failure	23 (7.9)	
stroke	18 (6.2)	
obesity	14 (4.8)	
solid organ transplant	6 (2.1)	
liver cirrhosis	4 (1.4)	
HIV	1 (0.3)	
bone marrow transplant	1 (0.3)	
Radiology (admission X-ray/CT)		
normal appearance	82 (28.1)	
consistent with viral pneumonitis	126 (43.2)	
other^b^	84 (28.7)	
Laboratory data on admission		
neutrophil/lymphocyte ratio	3.9	2.4–6.9
creatinine, mmol/L	81	64.3–108
alanine aminotransferase, IU/L	27	18–43
C-reactive protein, mg/L	44	16.2–122
d-dimer (<0.5 ng/mL normal)	0.615	0.41–1.17
troponin (<14 ng/L)	17	10–39
ferritin (ng/mL)	464	192–1039

a Other includes residential care facilities, recent hospitalizations and hospital-acquired patients.

b Atelectasis, lobar consolidation.

**Figure 1.**
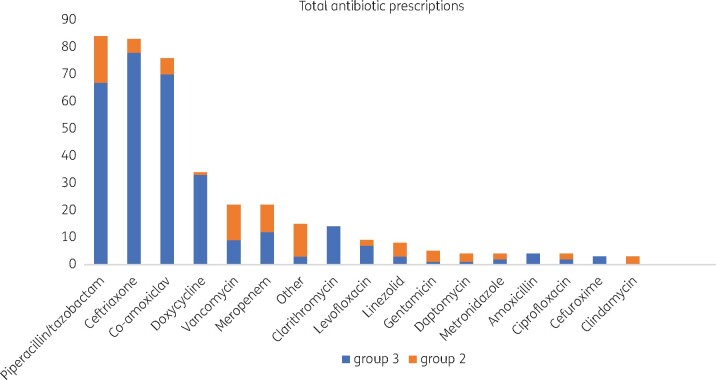
Total antibiotic consumption (Groups 2 and 3).

In total, 229 (78.4%) patients admitted with COVID-19 received antibiotics (excluding azithromycin). IV antibiotics were commenced in 66.1% of all patients admitted. The most common antibiotics prescribed were piperacillin/tazobactam (*n *=* *84 prescriptions), ceftriaxone (*n *=* *83 prescriptions) and co-amoxiclav (*n *=* *76 prescriptions), Figure 1. Local antimicrobial prescribing guidelines for community-acquired pneumonia (CAP) adopt CURB-65 scoring and advise use of either co-amoxiclav or amoxicillin with clarithromycin depending on severity score. In essence neither piperacillin/tazobactam nor ceftriaxone are adopted in the guideline. It must be noted that azithromycin was used in 140 patients ‘off-label’ in combination with hydroxychloroquine based on poor evidence for its use as a directed therapy for treatment of COVID-19. As its use in this role is not solely as an antibacterial agent it was excluded from antimicrobial analysis within the three subgroups. The cohort of 292 was divided into three sub-cohorts based on antimicrobial prescribing and those considered to have confirmed bacterial infection.

### Group 1 results

Group 1 included 63 (21.6%) patients, none of whom received antibiotics (excluding azithromycin). The median age was 44 years (IQR 28–58). No patients died and outcomes including median length of stay (6 days versus 11 Group 3, 28 Group 2), complications (6 versus 41 Group 3, 30 Group 2), high dependency care (2 versus 20 Group 3, 10 Group 2), re-admissions (5 versus 19 Group 3, 3 Group 2) and deaths (0 versus 29 Group 3, 9 Group 2) were best in this group, Table 2.

**Table 2.** Secondary outcomes

Interventions/outcomes	Total	No antibiotics (Group 1)	Empirical antibiotics for pneumonia (Group 3)	Antibiotics for proven bacterial infection (Group 2)	*P* value
*N* (%)	292 (100)	63 (21.6)	194 (66.4)	35 (12)	
COVID-19 directed, *n* (%)					
hydroxychloroquine + azithromycin	140 (48)	11 (17.5)	111 (57.2)	18 (51)	
remdesivir	1 (3.4)	—	1 (0.5)	—	
lopinavir/ritonavir	30 (10.3)	6 (9.5)	24 (12.4)	—	
tocilizumab	1 (3.4)	—	—	1 (2.9)	
Antibiotics, *n* (%)					
antibiotics—any indication	229 (78.4)	—	194 (100)	35 (100)	
commenced IV	193 (66.1)	—	165 (85)	32 (91.4)	
duration, days, median (IQR)	7 (5–10)	—	7 (5–10)	14 (10–20)	<0.001
Oxygen requirements					
duration of hypoxia, days, median (IQR)	1 (0–8)	0 (0–0)	4 (0–9)	8 (0–20)	<0.001
maximum respiratory support, *n* (%)					
HFOT	38 (13)	—	33 (17)	5 (14.29)	
NIV	19 (6.5)	—	16 (8.2)	3 (8.6)	
MV	20 (6.8)	1 (1.6)^b^	10 (5.2)	9 (25.7)	
ECMO	2 (0.68)	^—^	1 (0.5)	1 (2.9)	
Outcomes					
length of stay, days, median (IQR)	11 (6–20)	6 (3–12.5)	11 (7–18)	28 (16–45)	<0.001
complications per group, *n* (%)^a^	68 (23.4)	6 (9.5)	41 (21.1)	30 (85.7)	
CDI, *n* (%)	2 (0.68)	—	—	2 (5.7)	
high dependency care, *n* (%)	32 (10.2)	2 (3.2)	20 (10.3)	10 (28.6)	
readmission, *n* (%)	23 (7.9)	5 (7.9)	19 (9.4)	3 (8.6)	
death, *n* (%)	38 (13%)	—	29 (14.5%)	9 (25.7%)	

HFOT, high-flow oxygen therapy; NIV, non-invasive ventilation; MV, mechanical ventilation; ECMO, extracorporeal membrane oxygenation; CDI, *C. difficile* infection.

a Venous thrombo-embolus, acute kidney injury, arrhythmia, electrolyte disturbance, stroke, acute coronary syndrome, seizure, gastrointestinal bleed, decompensated heart failure.

b Status epilepticus.

### Group 2 results

Group 2 included 35 (12%) patients with objective evidence of bacterial infection of any cause, median age was 66 years (IQR 59–84). The most common infections were urinary tract infection including urosepsis (*n *=* *7), pneumonia including hospital associated or ventilatory associated (*n *=* *7), venous catheter-associated infection (*n *=* *7). *Escherichia coli* (*n *=* *5), MSSA (*n *=* *5), and *Staphylococcus epidermidis* (*n *=* *4) were the most common organisms identified. A spectrum of other infections was found: bacteraemias, *Clostridioides difficile* infections, skin and skin structure infections and neurosyphilis, amongst others. The incidence of infections and identified pathogens can be seen in Table S1 (available as Supplementary data at *JAC-AMR* Online). Longer antibiotic courses were seen in Group 2. Increased mortality, length of stay, need for mechanical ventilation, complication rate, and higher proportion of IV antibiotic use were also seen in this group, Table 2. Of those with pneumonia in this group the majority were male [*n *=* *4/7 (57.1%)], with a median age of 64 years (range 55–83); all received IV antibiotics with a median duration of 10 days (range 3–20) and a total median duration of 17.5 days (range 10–21). These pneumonia patients as a subgroup were oxygen dependent for longer (median 20 days, IQR 14–29.5) and were more likely to need intubation [*n *=* *4/7, (57%)] than the Group 2 cohort as a whole.

### Group 3 results

Group 3 included 194 (66.4%) patients who were prescribed antimicrobials empirically for pneumonia without positive microbiological investigations of other supportive evidence of bacterial infection like raised procalcitonin. Median age was 64 years (IQR 47–77) in this group.

The majority of individual antibiotic courses (309 of 394) were administered in this group, Figure 1. Linear regression was performed to determine variables that are associated with prescribing antibiotics at the time of antibiotic commencement, and variables that may be driving continuation of empirical antibiotics. For commencement of IV antibiotics on admission, higher qSOFA (*P *=* *0.016), C-reactive protein (CRP) (*P *=* *0.024), fever >37.5 °C (*P *=* *0.011) and age (*P *=* *0.023) were variables found to be significant, Table 3. None of the above factors was associated with duration of antibiotic course but duration of hypoxia was strongly associated with longer antibiotic course (*P *<* *0.001) as was maximum respiratory support requirement (*P *=* *0.013), Table 3.

**Table 3.** Logistic regression analysis of antimicrobial start and duration in Group 3: presumed pneumonia group, *N *=* *194

Independent variables on admission	*P* value	Exp (B)	95% CI
Dichotomous logistic regression—dependent variable: antibiotic start or not on admission			
hypoxia^a^	0.154	0.392	0.108–1.421
qSOFA	**0.016**	2.376	1.171–4.820
respiratory rate	0.086	0.864	0.731–1.021
heart rate	0.921	1.001	0.978–1.025
C-reactive protein	**0.024**	0.985	0.972–0.998
age	**0.023**	0.971	0.946–0.996
radiography	0.853	0.945	0.522–1.713
NLR	0.285	0.920	0.790–1.072
R^2=^0.491	**<0.001**		

Independent variables	*P* value	Std β coefficient	95% CI

Multivariable logistic regression—dependent variable: total antibiotic duration			
qSOFA	0.967	−0.03	−0.899–0.862
days of hypoxia	**<0.001**	0.586	0.182–0.299
maximum respiratory support^b^	**0.013**	0.185	0.081–0.665
age	0.417	0.053	−0.018–0.44
C-reactive protein	0.053	0.123	0.00–0.013
** **R^2^^**=**^0.308	**<0.001**		

Significant differences are highlighted in bold.

a SpO_2_ 94% or any supplemental O_2_ requirement.

b High-flow oxygen, non-invasive ventilation, mechanical ventilation, extra-corporeal membrane oxygenation.

Within Group 3, the median duration of an antibiotic course was 7 days (IQR 5–10). Of this group, 28 (14.4%) patients had an escalation of antibiotic therapy (switch from an oral agent to IV, or broadening of antimicrobial coverage), three of whom had two levels of escalation when perceived to be failing response to therapy (Table S2). The median time to escalation was 4 days (IQR 3–6). Twenty-seven (14%) patients in Group 3 had de-escalation of therapy (switch from IV to oral or narrowing of antimicrobial spectrum). The median time to de-escalation was 5 days (IQR 3–7). Within Group 3, 139 (71.6%) patients had neither escalation nor de-escalation of antibiotic therapy, the median antibiotic course duration for these individuals was 7 days (IQR 5–8).

## Discussion

In this study we have shown that there were high levels of antimicrobial consumption during the first wave of the pandemic in hospitalized COVID-19 patients as 229 (78.4%) of inpatients in this study received antibiotics. Early in the pandemic similarly high levels were also seen in international published cohorts at the time, particularly in China.^13–15^ A lack of understanding of secondary infection with COVID-19, and former experience with influenza for which most hospitalized patients receive antibiotics,^16–19^ most likely led to widespread international use of antibiotics at that time. It has been shown with time that bacterial coinfection with COVID-19 is low; a meta-analysis of 3834 patients showed only 7% of hospitalized patients develop secondary infection.^4^ Interestingly, a review of postmortem studies of 621 patients (from 75 studies) found bacterial pneumonia was a histological feature in 200 (35%) of cases, 50 (25%) of which had recoverable bacteria, representing 8% of the overall cohort. Most commonly *Acinetobacter baumannii, S. aureus, Pseudomonas aeruginosa* and *Klebsiella pneumoniae* were found. Pneumonia was by far the most common infection identified on postmortem (>95%).^5^ Given death is a certain marker of severity of COVID-19, bacterial infection rates are low and dominated by pneumonia.

In this study we have shown that clinical severity factors were most important in provider decision to prescribe IV antibiotics at the time of commencement; higher qSOFA, raised temperature and higher CRP were all significant (*P *<* *0.05). This is not entirely surprising as these factors are also indicative of bacterial sepsis, and in some instances SARS-CoV-2 positivity may have been suspected but not confirmed at the time of assessment. Interestingly, despite early commencement, de-escalation of antibiotics only took place in 31 (16%) of the presumed pneumonia cohort (Group 3) at 5 days. The majority of patients remained on IV antibiotics for a 7 day course. Duration of hypoxia and maximum level of respiratory support were the only identifiable factors that reached significance in determining antimicrobial duration. In retrospect, the majority of these patients did not have bacterial infection; the usual markers of infection like clearance of cultures, defervescence and resolving neutrophilia may not present in these patients, so hypoxia may have acted as the surrogate objective marker for prescribers in decisions surrounding escalating, continuing, de-escalating and stopping antibiotics. Maximum oxygen requirement was also found to be a significant factor (*P *=* *0.024) for duration of antibiotic courses in hospitalized patients in another Irish cohort of 117 patients, 72% of whom received antibiotics, with just 6% confirmed bacterial infection.^20^

Antimicrobial prescribing behaviour in the hospital setting is complex. Despite a rapidly evolving understanding of bacterial infection rates with COVID-19 this may not translate into good antimicrobial stewardship practices. One study examining prescriber knowledge and practice for CAP found there was a mismatch of these two factors: 84% of respondents in an audience response teaching session demonstrated correct guideline-based antimicrobial choice, yet in practice only 62.5% of prescribers subsequently made the correct choice. Prescriptions tend to be overly broad in antibacterial coverage.^21^ Studies have shown that prescribing can be dominated by cultural norms; prescribers are unlikely to interfere with decisions of their peers or more senior firm members.^22^ Future antimicrobial stewardship services could benefit from adopting behavioural science techniques to develop robust multifaceted programmes.^23–25^ These will likely be needed more than ever to reduce AMR; the consequences of the current pandemic have yet to be determined but may stretch well into the ‘post COVID-19’ era.^26^

One facet to antimicrobial stewardship that has not been addressed is the use of antifungal therapy, particularly in patients admitted to the ICU. In total, only one patient had proven *Candida* CVC-associated infection yet many of these patients received antifungals. Outbreaks of *Candida auris* have been described in hospital ICUs caring for COVID-19 patients.^27^^,^^28^ This additional facet adds to the complexity of the antimicrobial fallout from COVID-19.

Regarding secondary outcomes of the study, worse outcomes like length of stay, increased complication rate, need for intensive care and increased mortality compared with the other two groups were seen in Group 2 (Table 2). This indicates there is an association between infection and adverse outcomes, although a causal relationship is not being described as those with severe COVID-19 may have immune dysregulation and pre-disposition to infection as an endpoint, as is ICU admission, mechanical ventilation and death. In the setting of severely unwell and deteriorating patients early broad-spectrum antimicrobials are essential; promotion of sepsis surveillance and action strategies like sepsis-6 to mitigate the negative impact of bacterial sepsis on these already unwell patients should be an ongoing aspect to care of patients hospitalized with COVID-19.^29^

This study has a number of limitations. It is a single-centre, retrospective study and is therefore prone to bias. The sample size of 292 is relatively small in size. There may be variables we have not measured in this study that are driving antimicrobial decision-making for these patients. A prospective study would be useful in determining the change in antibiotic prescribing practices over time in the context of updated antimicrobial guidelines and AMS service practice. Interview or questionnaire-based studies exploring prescriber decision-making in hospitalized patients with COVID-19 would also be highly insightful. In this study we can only speculate behaviour based on observations of practice retrospectively. Another limitation of the study is the accuracy of sub-categorization based on the presence of true bacterial infection and difficulties surrounding HAP, VAP and true CVC infections. If patients were given a diagnosis of one of the above with documentation in the medical notes and had supportive microbiological cultures and radiological findings, the authors of this study did not scrutinize these diagnoses as to whether they were completely guideline compliant. In this study we assume that antimicrobial choice at the time of diagnosis of a specific infection is no longer empirical in these cases and choice made thereafter is driven by the respective guidelines for individual infections. Conversely some patients in Group 3 may have been under investigated due to resource limitation during the initial surge of COVID-19 admissions and unmeasured barriers including the logistics of acquiring and transporting samples to the laboratory. These patients may have been found to be culture positive had microbiological sampling been complete and therefore been categorized in Group 2.

### Conclusions

Empirical prescribing of IV antibiotics in hospitalized COVID-19 patients is associated with clinical severity markers like higher qSOFA, fever, older age and raised CRP. Markers of respiratory compromise including duration of hypoxia and maximum respiratory support may be drivers for longer antimicrobial courses. Patients considered to have true bacterial infection had worse outcomes in this study (Group 2). The lack of accurate diagnostics for bacterial infection in COVID-19 and over prescription of antibiotics is likely to have a knock-on effect on the potentially larger and longer lasting global health crisis that is antimicrobial resistance.

## Supplementary Material

dlab085_Supplementary_Data
