# Supplementary material for: Antibiotic prescribing patterns in patients hospitalized with COVID-19: lessons from the first wave
Source: JAC Antimicrob Resist. 2021 Jun 30;3(2):dlab085. doi: 10.1093/jacamr/dlab085 (PMC8242139; doi:10.1093/jacamr/dlab085)
Supplement: dlab085_Supplementary_Data [file dlab085_supplementary_data.docx]

| Infection | N | organisms |
| --- | --- | --- |
| Urinary tract infection including urosepsis | 9 | *e.coli x3, proteus mirabilisx2, e.coli* (ESBL) x2, *klebsiella pneumoniae x2* |
| Pneumonia including ventilatory and hospital associated pneumonia | 8 | *e.coli, proteus mirabilis, Citrobacter koseri, pseudomonas aeruginosa,* MRSA x2, e*nterobacter aerogens* |
| Venous catheter infection | 7 | MSSA x1*, staphylococcus epidermidis x4, enterococcus faecium x2* |
| Clostrium difficile infection | 3 | *Clostridium difficile* |
| Bacteremia | 5 | MSSAx2, *pseudomonas aeruginosa, e.coli, bacteroides fragilis* |
| Septic arthiritis (with bacteremia) | 1 | Group C/G streptococcus |
| Cellulitis (including infected peripheral ulcers) | 2 | MSSA, *pseudomonas aeruginosa* |
| Osteomyelitis/psoas abscess | 1 | MSSA |
| Pneumocystis pneumonia | 1 | ­*pneumocystis jirovecii* |
| neurosyphilis | 1 | *treponema pallidum* |

**Supplementary data**

**Table S1.** Confirmed infection – Group 2 patients

**Table S2.** Antimicrobial switch in group 3 patients N=194

| **Antibiotic switch of therapy** | **n** |
| --- | --- |
| **One-step escalation** | **N=25** |
| - doxycycline to pip-tazobactam | 1 |
| - ceftriaxone to co-amoxiclav | 2 |
| - ceftriaxone to pip-tazobactam | 8 |
| - co-amoxiclav to co-amoxiclav (po to iv) | 1 |
| - co-amoxiclav to pip-tazobactam (iv to iv) | 6 |
| - co-amoxiclav to pip-tazobactam (po to iv) | 1 |
| - pip- tazobactam to meropenem | 3 |
| - pip- tazobactam to meropenem+vancomycin | 2 |
| - pip- tazobactam to meropenem+linezolid | 1 |
|  |  |
| **Two-step escalation** | **N=3** |
| - ceftriaxone to pip-tazobactam to meropenem | 2 |
| - ceftriaxone to ceftriaxone+vancomycin to meropenem+linezolid | 1 |
|  |  |
| **De-escalation of therapy** | **N=27** |
| - ceftriaxone to co-amoxiclav (iv to po) | 15 |
| - co-amoxiclav to co-amoxiclav (iv to po) | 7 |
| - pip-tazobactam to co-amoxiclav (iv to po) | 4 |
| - meropenem to co-amoxiclav (iv to po) | 1 |
